# Editorial: Using rootstocks in crops and fruit trees to mitigate the effects of climate change and abiotic stress

**DOI:** 10.3389/fpls.2024.1479317

**Published:** 2024-09-09

**Authors:** Juan-Pablo Martínez, Boris Sagredo, María Ángeles Moreno

**Affiliations:** ^1^ Plant Physiology and Molecular Biology Laboratory, Instituto de Investigaciones Agropecuarias (INIA-Rayentué), Rengo, Chile; ^2^ Department of Pomology, Estación Experimental de Aula Dei - Spanish National Research Council (CSIC), Zaragoza, Spain

**Keywords:** rootstock, abiotic stress, fruit trees, climate change, crops, heat stress, iron-clorosis deficiency

## Background

Climate change (CC) is one of the most important challenges in feeding the world’s growing population. CC, and consequently salinity, drought, waterlogging, and low or high temperatures known as abiotic stress (AS), is having dramatic consequences on agriculture worldwide (www.fao.org). Currently, approximately 40% of the Earth’s surface is land under drought and salinity stress, and it is predicted that this will increase to 50% by 2025 (United Nations, 2014). AS affects crop growth and production, reducing yields by more than 50% (Boyer, 1982; Pessarakli, 2019), including horticulture, crops, and vegetables of major importance to world economies. Therefore, plants with improved stress resistance or tolerance are desirable to support agricultural productivity and environmental sustainability. One of the main limitations of modern varietal improvement that has been focused on quality traits is to satisfy consumer demand more than adaptive traits that allow plant development under adverse conditions, which are found in a package of genes of wild populations. The tolerance and adaptation present in such genetic material can be used directly through the root system after minimizing the problems of genetic graft incompatibility. It represents a faster alternative to obtaining cultivars through conventional breeding, which requires a long time, and sometimes, the genes of interest cannot be introduced.

To cope with the above mentioned limiting factors, the use of grafted plants on tolerant rootstocks in crops and fruit trees has been recommended as the best sustainable strategy. The advantage of using rootstocks allows growers to maximize profits, especially when working under stress conditions, and reduce inputs such as water and fertilizers. In this sense, a practical alternative for breeders seems to be the introduction of genes associated with AS tolerance to rootstocks, thereby converting a sensitive cultivar into a tolerant one, maintaining all the highly valuable characteristics that current cultivars possess. The advantage of using rootstocks is that they rapidly accelerate the adaptation of crops and trees to abiotic stresses. The screening and obtaining of higher salt-tolerant rootstock genotypes would undoubtedly be accelerated if physiological traits related to AS were identified. It has been experimentally proved that the use of tolerant cultivars as rootstocks increases the yield of susceptible cultivars to salinity in tomato (He et al., 2009; Martínez et al., 2024), drought in apple (Tworkoski et al., 2016; Li et al., 2024), high temperature in tomato (Lee et al.), and mineral toxicity in citrus (Yang et al., 2023), among others. Therefore, the aim of the present Research Topic is to show the physiological and molecular aspects of tolerance to different types of abiotic stresses (drought, salinity, root asphyxia, iron chlorosis, and high or low temperatures) associated with the use of rootstocks in crops and fruit trees as a strategy to counteract these stresses. Selection of rootstock is nevertheless a challenging task and requires combining numerous root-related traits. In this context, the tomato wild relatives *Solanum pennellii* and *Solanum peruvianum* are known to be drought tolerant, and their responses to heat stress have been studied in this topic, especially when used as high temperature-tolerant rootstocks for grafting (Lee et al.). These authors showed that the wild relative rootstock *S. peruvianum* was effective in enhancing the thermotolerance of scion tomato seedlings, showing potential as breeding material for the introgression of heat-tolerant traits in interspecific tomato rootstocks. In contrast, in the tomato wild relatives (*Solanum chilense* and *S. peruvianum*), numerous data are available to date for some closely related wild halophyte species (Tapia et al., 2016; Gálvez et al., 2012; Albacete et al., 2009; Bigot et al., 2022, 2023a), and interspecific crosses with cultivated *Solanum lycopersicum* (Asins et al., 2013; Bigot et al., 2023b; Martínez et al., 2024) or introgression lines from wild species (Julián et al., 2013) have been successfully performed under saline stress. It has also been observed in different studies that rootstocks used in both vegetables and fruit trees affect plant vigor and water status. By evaluating carbon (δ^13^C), oxygen (δ^18^O), and nitrogen (δ^15^N) isotopic composition, relationships with carbon assimilation, water relations, and shoot growth can be established. Casagrande Biasuz and Kalcsits observed that apple rootstocks affected scion shoot growth, which was consistent with known vigor levels for the four studied rootstocks and consistent between the two scion cultivars. In addition, changes in water relations influenced by rootstock genotype significantly affected leaf, stem, and root δ^13^C, δ^18^O, and δ^15^N. Lower δ^13^C and δ^18^O were inconsistently associated with rootstock genotypes with higher leaf, stem, and root vigor. Another important characteristic to be considered in rootstock selection is its capacity for nutrient uptake and how this is affected by the environmental conditions in which it develops. Related to this fact, Xie et al. demonstrated the key role of rootstocks in nutrient uptake and environmental adaptation in apple. In this work, they found that strategies for adaptation to N and/or P deficiency in apple included inhibition of the above-ground growth, enhancing root development and improving the root-to-shoot ratio. Alterations to root architecture under N and/or P deficiency were associated with several factors, including a) increased partitioning of total N and total P in the root; b) contribution of H^+^ efflux to the uptake of NO_3_^−^ and P, leading to an external decrease in pH, and thus root cell wall extension; and c) altered root cell wall components. This topic is also interested in different aspects of genomics and molecular biology that will help to elucidate the genetics of tolerance to these stresses. Guajardo et al. elucidated the genetic basis of leaf iron-chlorosis tolerance using the phenotypic segregation of a peach–almond progeny to iron-chlorosis stress, along with the quantitative trait loci (QTLs) responsible for leaf chlorosis at the EEAD-CSIC *Prunus* rootstock breeding program. From the initial mapping population, 131 individuals were selected for their phenotypical characterization with soil plant analysis development (SPAD) measurements of plants grown in the field, exhibiting great variability. Significant QTLs associated with tolerance to iron chlorosis were found in linkage groups (LGs) LG1, LG5, LG7, and LG8. The significant QTLs detected in LG5 and LG7 have not been associated with this abiotic stress before in *Prunus*. The aim is to apply this information to develop tools and biotechnological technologies to improve the performance of plants (scions) grafted on rootstocks tolerant to abiotic stresses.

Most of the genes for adaptation to different types of stress are found in wild populations, which have undergone less selection than commercial varieties. These adaptive genes are difficult to transfer to modern cultivars because, among other reasons, the selection and filtering process often loses the “gene package” suitable for adaptation to the changing environment by selecting genes for productivity and fruit quality of the plant at harvest and post-harvest. The use of hybrid rootstocks of the same species or interspecific crosses does not require the high degree of screening and selection that disarming adaptive gene packages represent but can be used directly by ensuring good graft compatibility with the scion. New biotechnological tissue culture techniques allow clonal propagation of superior hybrid genotypes. A better knowledge of the mechanisms underlying this adaptation, as shown in these studies, will allow a better response to adapt crops to abiotic stresses accentuated by climate change. Other issues related to the use of rootstocks, such as their adaptation in interaction with rhizosphere microorganisms, are lines of research that will allow a better understanding of the adaptation mechanisms used by plants in the environments in which they have been developed and thus use them for the benefit of agriculture and food security in the context of climate change.
